# Attentional Impairments and Neural Compensation in Adolescents with High Social Anxiety Traits: A Combined ERP and Functional Connectivity Study

**DOI:** 10.3390/jintelligence14040051

**Published:** 2026-03-25

**Authors:** Wenqing Lin, Xinmei Deng

**Affiliations:** 1 School of Psychology, Shenzhen University, Shenzhen 518060, China; 18823100022@163.com; 2 Key Laboratory of Brain Cognition and Emotional Health of Guangdong Higher Education Institutes, Shenzhen University, Shenzhen 518060, China; 3 Shenzhen Key Laboratory of Affective and Social Cognitive Science, Shenzhen University, Shenzhen 518060, China

**Keywords:** adolescents with social anxiety, social information processing, EEG

## Abstract

Adolescence is a key period of significant physiological and social development, during which social anxiety symptoms often emerge and can impact academic and social functioning. Social anxiety disorder (SAD) involves heightened sensitivity to social cues and impaired social information processing, potentially contributing to persistent anxiety symptoms. However, research exploring the neural mechanisms of social information processing in adolescents with social anxiety remains limited. The investigation employed a facial dot-probe paradigm combined with EEG measurements to assess differences in attentional processing and neurophysiological activity between two adolescent groups: a high-social-anxiety (HSA) group (N = 27) and a low-social-anxiety (LSA) group (N = 18). Results showed (1) there was a significant reduction in P2 amplitudes in the HSA group compared to the LSA group. (2) A significant negative correlation between the disengagement index (DI) and P2 amplitude was found. (3) Weaker functional connectivity in the theta band was found in the HSA group. (4) In the graph theory analysis, the HSA group exhibited significantly higher node efficiency across various frequency bands compared to the LSA group. The findings suggest that socially anxious adolescents have impaired attentional control toward social cues. This difficulty may reinforce their anxiety symptoms over time.

## 1. Introduction

Adolescence, commonly defined as the period between ages 13 and 18 years according to MeSH criteria (National Library of Medicine, n.d.), is a critical phase of neurological and social development. The social, structural, and cultural contexts in which people are situated influence how these developmental changes unfold (Choudhury et al., 2023). Within this complex developmental landscape, adolescents exhibit increased sensitivity to social contexts and heightened vulnerability to impaired social interaction patterns (Topel et al., 2021), which may contribute to the rise in social anxiety symptoms observed during this stage. Social anxiety disorder manifests through excessive concern regarding others’ evaluations, resulting in unease and a tendency to avoid social encounters or performance scenarios (Miers & Warner, 2023). As a result, adolescents with high levels of social anxiety have severe scholastic and interpersonal challenges, as well as a greater risk of developing comorbid psychological illnesses (Vogel et al., 2021). Smith et al. (2020) discovered that adolescent social anxiety is linked to different brain processing patterns associated with continued maturation of the striatum and temporal lobe structures (middle and superior temporal gyri). When adolescents under the age of 13 experience higher levels of social anxiety, their brain sensitivity to unpredictable negative feedback increases while their responses to predictable positive input decrease. However, the brain mechanisms that underpin social processing in adolescents with high social anxiety remain poorly understood. Consequently, the current study seeks to investigate differences in neural responses between adolescents with social anxiety and those in a comparison group during the processing of social stimuli in order to deepen our understanding of the neural processes associated with social anxiety at this critical developmental stage.

### 1.1. Social Anxiety

Social anxiety disorder (SAD) is a chronic mental condition characterized by excessive dread in social circumstances and persistent worry about negative assessment by others (Salari et al., 2024). The tendency to focus disproportionately on emotional cues is a basic component of social anxiety, significantly impacting its etiology and long-term manifestation (Jiang et al., 2024). Experimental findings appear to suggest that persons with social anxiety tend to exhibit what might be characterized as maladjusted affective reactivity patterns that seem to extend across stimulus valence from negative to positive social information. Evans et al. (2016) appear to have uncovered what seems to be abnormal reactivity to negative stimuli in socially anxious individuals. What Yu et al. (2014) reported tends to suggest similar maladaptive reactions to positive stimuli. What appears particularly significant about these findings is that individuals with higher social anxiety symptoms ostensibly devote more attention to the eye region when processing information about social faces than those with lower social anxiety symptoms. Within this broader analytical framework, this might indicate that people with higher degrees of social anxiety are seemingly more sensitive to social face cues (Gilboa-Schechtman & Shachar-Lavie, 2013; Song et al., 2023). What the evidence appears to reveal is that their enhanced attentional focus on the eye region may represent a greater sensitivity and response to social cues, particularly those indicating possible threat or negative appraisal. As a result, it is worth investigating how socially anxious adolescents direct their attention and process social information.

Research shows that individuals with social anxiety disorder have stronger emotional reactions when they see different types of facial expressions. People with this kind of sensitivity may better recognize emotions at lower levels of intensity and are more accurate at doing so (Cui et al., 2021). Individuals with social anxiety are also more likely to think that neutral facial expressions are threatening and to think that unclear emotions mean negative things (Bell et al., 2011; Yoon et al., 2014).

Event-related potential (ERP) studies have indicated that individuals with social anxiety interpret emotional stimuli differently than individuals without it. Individuals with anxiety had a significantly lower P2 amplitude, which shows that they interpret emotional information visually more selectively at an early stage than those who do not have anxiety (Frenkel & Bar-Haim, 2011). According to studies, individuals who had more social anxiety showed far lower P2 amplitudes when they were looking at emotional facial expressions than those with less social anxiety (Rossignol et al., 2013; Bechor et al., 2019). This decrease in P2 amplitude may indicate that socially anxious individuals pay less attention to emotional stimuli. Extending this line of evidence to the studies of emotion regulation, L. Yuan et al. (2014) found that during face viewing, individuals with low social anxiety traits exhibited larger P2 amplitudes compared to those with high social anxiety traits. This reduction in P2 amplitude may reflect impaired early-stage facial information integration in individuals with high social anxiety.

Research on functional connectivity has found impairment of the brain networks of people with social anxiety. Research has suggested that individuals with a greater amount of social anxiety have significantly low levels of functional connections between the anterior amygdala and the areas of the medial orbitofrontal cortex and the posterior cingulate cortex/precuneus (Hahn et al., 2011). A total of 32 people with generalized social anxiety disorder (gSAD) and 32 matched healthy controls (HCs) participated in an EEG study by Xing et al. (2017). Analysis of the resting-state functional connectivity using the weighted phase lag index (WPLI) revealed an increased theta-band coherence in the mid-frontal region in the gSAD group compared to HCs, indicating hyper-connectivity during rest. The findings of previous studies show that social anxiety disorder is characterized by an increase in functional connectivity between the left amygdala and a variety of cortical regions (Mizzi et al., 2024). These regions participate in various processes, including face recognition, emotion regulation, attention processing, and self-referential cognition. On the contrary, functional connectivity between the right amygdala and the middle temporal gyrus was found to be lower in these persons (Jung et al., 2018).

Studies in the field of graph theory have identified the global characteristics of the brain networks of individuals who are socially anxious. Recently, research has indicated that patients with social anxiety disorder exhibit more efficient nodes and increased connectivity in their neural networks than healthy individuals (Al-Ezzi et al., 2023). According to Birk et al. (2019), those who suffer from social anxiety disorder also perceive more danger in social situations and are more sensitive to unfavorable opinions. The higher sensitivity to such stimuli in individuals with social anxiety may be explained by the fact that the neural networks of such individuals are more intermeshed and seek to pay closer attention and process the stimuli of a social nature.

### 1.2. Social Anxiety in Adolescence

Adolescence is the phase when the development of social anxiety disorder frequently occurs (Chiu et al., 2021). The brain undergoes intense stages of maturation in the form of brain circuitry during adolescence, which entails synaptic pruning and myelination (Natu et al., 2019). These developmental changes enhance structural integrity and functional connection between the subcortical and the cortical areas (Heller et al., 2016). The results of research show that comparatively poorly developed capacities of emotional regulation, coupled with the increased capacity to accommodate various perspectives within a context, increase the possibility of specific neurocognitive risk factors of social anxiety manifesting themselves as behaviors associated with social anxiety during this phase of development (Haller et al., 2015). Most researchers have attributed social anxiety to disorders of the amygdaloid fear circuit. According to Freitas-Ferrari et al. (2010), the front-limbic circuitry, which includes the amygdala, anterior cingulate cortex (ACC), ventromedial prefrontal cortex, and dorsolateral prefrontal cortex, is affected in those who have social anxiety disorder. Battaglia et al. (2011) found that adolescents with social anxiety disorder have more sensitive amygdalae and different neural responses in the ACC, striatum, medial prefrontal cortex (mPFC), ventrolateral prefrontal cortex (vlPFC), and insula when they perceive negative social cues like facial expressions. This is in line with what was said previously. Also, the fact that adolescents with high levels of social anxiety have lower structural covariance between the insula and orbital frontal cortex (OFC) may be a sign that their brains are not developing at the same rate, which could lead to problems with regulating emotions, less motivation to socialize, and more avoidance of social situations (Liu et al., 2020). This disturbance in developmental coordination may be a key neurobiological reason why adolescents with social anxiety symptoms keep having them.

In adolescents with social anxiety traits, facing another person who is looking back at them elicits increased arousal and a negative emotional response. It also induces frontal EEG asymmetry in brain activation and a concurrent tendency to avoid eye contact at the behavioral level. Conversely, adolescents without anxiety generally exhibit heightened arousal coupled with positive emotions when engaging in direct eye contact (Myllyneva et al., 2015). Thai et al. (2016) found a negative correlation between the P2 amplitude and the social anxiety level in a dot-probe study of behaviorally inhibited adolescents. Bechor et al. (2019) found that typically developing adolescents exhibited larger P2 amplitudes compared to those with anxiety disorders during a dot-probe task. Mao and colleagues (2020) discovered enhanced orbitofrontal-amygdala functional coupling in socially anxious adolescents compared to non-anxious peers. This increased connectivity may reflect compensatory regulatory mechanisms by the OFC to modulate amygdala hyperactivation during negative emotional processing. Longitudinal findings show that OFC gray matter volume and OFC-amygdala functional connection are neural predictors of future social anxiety severity (Mao et al., 2020). These findings indicate that adolescents with social anxiety have distinct brain responses to social stimuli when compared to their healthy peers. These immature neural networks could contribute to the continuation of social anxiety symptoms.

Taken together, behavioral and neuroimaging studies suggest that social anxiety in adolescents involves altered processing of social cues. However, the empirical findings remain complex and sometimes inconsistent. For example, while some dot-probe studies report vigilance toward threat faces, others find avoidance or null effects (Bantin et al., 2016). Moreover, investigations of functional brain networks have yielded mixed results, reporting both increased and decreased connectivity within fronto-limbic and default-mode circuits (Hahn et al., 2011; Al-Ezzi et al., 2023). Crucially, few studies have combined temporally precise electrophysiology with measures of large-scale network organization to provide an integrated view of these processes during adolescence.

### 1.3. The Present Study

Existing neuroimaging studies on social processing in socially anxious adolescents have yielded inconsistent results (Rauschenbach et al., 2024). These inconsistencies emphasize the need for additional research to clarify the underlying brain mechanisms that govern how adolescents with social anxiety process social stimuli. The dot-probe task has been frequently used to examine attentional biases in individuals with social anxiety (Torrence & Troup, 2017), but the processing of facial stimuli, which may allow for a more sensitive measurement of biased processing, has yet to be fully investigated in this context. Given that facial expressions convey critical social cues, they hold particular significance for those with social anxiety, as these cues may signal potential negative evaluation by others (Bantin et al., 2016). The current study utilizes a facial dot-probe paradigm to examine neural correlates of social information processing in anxious adolescents. Extending prior findings that linked reduced P2 amplitudes to greater social anxiety symptoms in children (Thai et al., 2016) and consistent with evidence of attenuated ERP components in anxious adolescents during attentional tasks (Wauthia et al., 2022b), we predict significantly diminished P2 amplitudes in high versus low social anxiety adolescents. Also, functional connectivity and neural network transmission efficiency of different brain regions are examined and compared between adolescents with social anxiety and comparison adolescents. According to previous research, adolescents with higher levels of social anxiety may exhibit enhanced functional connectivity in brain regions associated with emotion regulation (Mao et al., 2020). Al-Ezzi et al.’s (2023) study revealed significant heightened connectivity between brain regions in social anxiety individuals, particularly through theta (4–7 Hz) and alpha (8–12 Hz) oscillations. Therefore, it is hypothesized that adolescents with higher social anxiety have stronger functional connectivity and higher neural network transmission efficiency than those with low social anxiety.

## 2. Materials and Methods

### 2.1. Participants

Adolescent volunteers were recruited from a local middle school through flyers for a study on emotion in China. The sample size required for this study was calculated using G*Power 3.1 software (Faul et al., 2007) with the following parameters: *F*-test, repeated-measures ANOVA, effect size *f* = 0.25, significance level *α* = 0.05, power (1 − *β*) = 0.8, number of groups = 2, and number of measurements = 2. At least 34 adolescents were required in total. We invited 54 participants to the study. Participants were divided into two groups based on their self-reported levels of social anxiety traits: a high-social-anxiety (HSA) group and a low-social-anxiety (LSA) group. This classification was derived from their scores on the Chinese version of the Social Anxiety Scale (La Greca et al., 1988), which has been employed in prior research with Chinese adolescents (Zhou et al., 2008). In line with prior studies (McGrath et al., 2016; Deng et al., 2022), individuals scoring 8 or higher were placed in the high-social-anxiety group, whereas those with scores below 8 were assigned to the low-social-anxiety group. All participants were screened to have no history of neurological disorders or other major psychiatric conditions. No formal clinical diagnosis of social anxiety disorder was obtained; thus, the study focuses on social anxiety traits within an adolescent sample. Family history of social anxiety disorder was not assessed. The study removed nine subjects due to technical issues and poor-quality EEG data. As a result, the final sample included 45 participants: 27 in the high-social-anxiety group (HSAs; 16 females and 11 males, aged 11–17 years, *M*_age_ = 15.89, *SD* = 1.41) and 18 in the low-social-anxiety group (LSAs; 4 females and 14 males, aged 11–18 years, *M*_age_ = 14.61, *SD* = 3.54). Eleven of the adolescents were the family’s only child. This variable was recorded for descriptive purposes but was not included in the primary statistical analyses. All participants were right-handed and had normal or corrected-to-normal vision. They had no prior history of current unstable medical illness, head injury, or neurological illness. After introducing the experimental procedure, the participants were asked to sign an informed consent form and complete a demographic questionnaire assessing age, gender, parental education level, and only-child status. Table 1 provides a summary of the two groups’ demographic attributes. Shenzhen University’s Institutional Review Board gave its approval to the experimental protocol for this study (Approval Number: SZU_PSY_2025_078). This research obtained institutional review board approval and adhered strictly to both national ethical regulations and the principles outlined in the Helsinki Declaration (1964) with all subsequent revisions. All procedures involving participants followed these established guidelines.

### 2.2. Design and Procedures

The study adopted a 2 (Group: HSA vs. LSA) × 2 (Condition: congruent vs. incongruent) mixed design. After being introduced to the experimental procedure, the participants were asked to sign an informed consent form and fill in demographic information. Then, they completed the dot-probe task.

### 2.3. Dot-Probe Task

**Figure 1:**
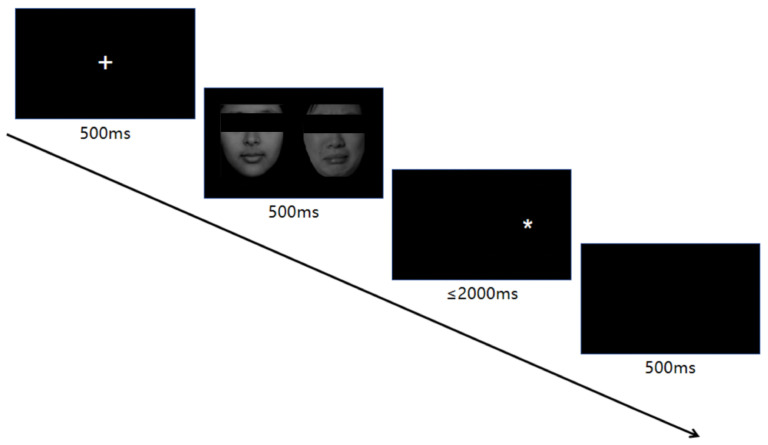
Illustration of the dot-probe task, presenting a congruent cue trial. “+” indicates the fixation point.

To assess attention bias, participants performed the dot-probe task (Figure 1), first designed by MacLeod et al. (1986). Following a 500 ms fixation display, participants viewed bilateral face pairs with negative emotional and neutral expressions from the Chinese Facial Affective Picture System (CFAPS). Equal numbers of male and female faces (500 ms) were subsequently masked by a probe stimulus (“*”) appearing for 2000 ms in either visual field. Participants were instructed to indicate the location of the probe (left or right) as quickly and accurately as possible by pressing the corresponding key. Keyboard responses (F = left, J = right) were collected during this period, with emphasis on both response speed and accuracy. Trials progressed regardless of the accuracy of the response, with an inter-trial interval of 500 ms.

The experiment comprised 100 trials, split into 3 blocks. A total of 40 trials were assigned to the congruent conditions (CCs) and incongruent conditions (ICs), with the remaining 20 trials dedicated to the neutral control conditions: (1) Congruent trials: In these trials, two faces were presented side by side. The probe appeared in the location of one of the faces. (2) Incongruent trials: In these trials, two faces were presented side by side. The probe appeared in the location of the other face. (3) Neutral trials: In these trials, two faces were presented side by side. The probe appeared randomly at the location of either the left or right face, regardless of whether the preceding condition was CC or IC. Each participant’s session took approximately 5 to 10 min.

### 2.4. EEG Recording and Data Analysis

#### 2.4.1. EEG Recording and Preprocessing

During the dot-probe task, continuous EEG recordings of the adolescents were obtained using a 32-channel portable EEG system (BrainAmp, Brainproducts GmbH, Gilching, Germany). Two electrodes were positioned on the left and right mastoids according to the international 10/20 system. The EEG data were captured at a sampling rate of 500 Hz, and electrode impedances were kept below 5 kohms.

The EEGLAB toolbox in MATLAB 2021b was used to examine the EEG preprocessing (Delorme & Makeig, 2004). Following established protocols (Schubring & Schupp, 2019), EEG data underwent offline processing, including average re-referencing and 1–40 Hz bandpass filtering. Artifact correction involved both ICA decomposition for ocular artifacts and manual inspection for gross motor artifacts. Data exclusion criteria comprised both behavioral errors in the dot-probe task and trials with amplitude excursions exceeding ±100 μV. Stimuli onset was set as the presentation of the face. EEG epochs were segmented 100 ms before and 500 ms after the stimulus onset. Following EEG preprocessing, 40 out of 50 trials, or more than 90% of the total number of trials, were valid segments. The short-time Fourier transform (STFT) was used to convert the EEG data following thorough preprocessing in Matlab.

#### 2.4.2. Behavioral Data

Task performance was calculated by attentional bias (AB) with the reaction time (RT). RT was assessed from when the stimulus began until the subject’s response. To improve the data’s reliability, all RT tests that deviated from the mean by more than ±2.5 standard deviations were not included in the study. To determine the attentional bias (AB) in all the subjects for each group, the disconnection index (DI), orientation index (OI) and bias index (BI) were calculated (Grafton & MacLeod, 2014, 2016; Hornung et al., 2019). These indices provided valid quantitative measures for determining differences in the task performance of different groups in the present study (see Appendix A).

#### 2.4.3. ERPs

Event-related potentials (ERPs) are scalp-recorded neural activations that are time-locked to specific events, offering millisecond-level temporal precision (Sur & Sinha, 2009). In the study of attentional bias, ERPs serve as a noninvasive method to examine covert attentional allocation (Judah et al., 2015) and can reveal biases that are not detectable through behavioral measures such as reaction times (Kappenman et al., 2014).

Based on previous research by Thai et al. (2016) and Revers et al. (2023), the mean amplitude of the P2 component measured at the Pz electrode was computed across the temporal window of 140–200 ms after the stimulus presentation. Before being analyzed, continuous EEG data were preprocessed with a bandpass filter that worked between 0.1 and 40 Hz. Artifact rejection excluded trials where the voltage changed by more than ±100 μV. Epochs from −100 to +500 ms relative to stimulus onset were extracted. Baseline correction was applied to the pre-stimulus interval (−100–0 ms). Based on previous research by Thai et al. (2016) and Revers et al. (2023), the mean amplitude of the P2 (Pz, 140 to 200 ms) was computed. Peak amplitudes within these time windows were identified and extracted using custom scripts in the EEGLAB toolbox, followed by visual inspection to verify the accuracy of component detection.

#### 2.4.4. Functional Connectivity

Lee et al. (2019) note that the phase locking value (*PLV*) is a nonlinear measure estimating pairwise functional connectivity. It measures phase synchronization of two nonlinear time-series biological signals, i.e., when recording EEG. According to Di Biase et al. (2023), there is noticeable synchronization in the different areas of the brain when the *PLV* are high. The Hilbert transform is used to get the instantaneous phase of EEG signals. Such a transformation enables one to express the signal in the complex space where scientists can determine the phase at any instantaneous moment. The *PLV* measures the constant of the phase variation between two signal frequencies. All the calculations of *PLV* between any two electrodes *i* and *j* were done as follows:

(1) $$PLV_{ij}=\left|\frac{1}{N}\sum_{t=1}^{N}\;e^{i(\phi_{i}(t)-\phi_{j}(t))}\right|$$

where *Φ_i_* (*t*) and *ϕ_j_* (*t*) represent the instantaneous phases corresponding to electrodes *i* and *j* at every discrete time point *t*. The parameter *N* represents the total number of time points or trials to determine synchronization. Phase locking value (*PLV*) is between 0 and 1; a value close to 1 indicates increased functional connectivity, while a value close to 0 indicates decreased functional connectivity. Functional connectivity analysis included frequency bands like the delta band (1–3 Hz), theta band (4–7 Hz), alpha band (8–13 Hz), beta band (14–30 Hz), and gamma band (31–50 Hz) (Kelsen et al., 2022; H. Wang et al., 2025). The GRETNA computational toolbox (J. Wang et al., 2015) was used to perform graph theoretical analyses on the complete connectivity dataset. The BrainNet Viewer platform (Xia et al., 2013) was also used to display the resulting networks.

#### 2.4.5. Graph Theory

The organization of graphs constructed to examine the structural and functional attributes is known as a graph network G (Sporns, 2013). Images were collected from the network associated with the nodes (the EEG electrodes) that observed activity of the different parts of the brain. The edges were determined based on statistical tests of weighted connections. These characteristics can be described in detail with the help of network analysis (Borsboom et al., 2021). Global measures refer to single quantities explaining the characteristics of the entire network. The metrics allow for a general overview of the network organization. Local measures, on the other hand, give each node a separate value. The procedure yields the length of that vector, which corresponds to the number of nodes in the net (Al-Ezzi et al., 2023). The method allows researchers to analyze each node and the global picture (Al-Ezzi et al., 2023). As regional network measures, we calculated node degree (*Dc*), node strength (*Ne*), node betweenness centrality (*Bc*), and node lateralization (*Nlp*).

(1) The number of edges incident on a node is *Dc*. The formula for the degree of a node in an undirected graph is

(2) $$Dc_{i}=\sum_{j=1}^{N}\;A_{ij}$$

Here, *A_ij_* is a part of the adjacency matrix. If there is an edge that links node *i* to node *j*, the value of *A_ij_* is 1. If no such edge is present, *A_ij_* is 0.

(2) *Ne* represents the sum of the weights of the links connected to a node. In a weighted graph, a node’s strength may be calculated using the following equation:

(3) $$Ne_{i}=\sum_{j=1}^{N}\;W_{ij}$$

In this regard, *W_i_* refers to an element of the weight matrix, representing the association level between node *i* and node *j*.

(3) *Bc* calculates the total fraction of shortest paths between all pairs of nodes that pass via a specific node. In this case, the following formula is applicable:

(4) $$Bc_{i}=\sum_{j\neq i\neq k}\;\frac{\sigma_{jk}(i)}{\sigma_{jk}}$$

In this formula, *σ_jk_* represents the total number of shortest paths between nodes *j* and *k*, and *σ_jk_* represents the number of those paths that go through node *i*.

(4) *Nlp* is a way to quantify how uneven the connections are between a node and the left and right hemispheres. The formula is

(5) $$Nlp_{i}=\frac{S_{i}^{L}-S_{i}^{R}}{S_{i}^{L}+S_{i}^{R}}$$

Here, $S_{i}^{L}$ represents the total connection strength between node *i* and nodes in the left hemisphere. $S_{i}^{L}$ represents the total connection strength between node *i* and nodes in the right hemisphere.

Network analysis within this research was performed by employing the GRETNA toolbox (J. Wang et al., 2015).

### 2.5. Statistical Data Analyses

(1) ERP: Separate 2 (Group: HSA vs. LSA) × 2 (Condition: congruent vs. incongruent) repeated-measures ANOVAs were conducted on P2 amplitudes.
(2) Correlation analysis: Pearson’s correlations were computed to assess the relationships between attention bias indices (bias index, orienting index, disengagement index), social anxiety symptoms, and the analyzed ERP components (P2 amplitudes).
(3) Functional connectivity analysis: (a) The *PLV* was computed to assess functional connectivity between brain regions. (b) To compare lingual L’-R’ amplitude differences between HSA and LSA groups, an independent-samples *t*-test was performed.
(4) Graph theory analysis: Graph theory analysis was conducted to examine the topological properties of brain networks, including node degree (*Dc*), node strength (*Ne*), node betweenness centrality (*Bc*), and node lateralization (*Nlp*). These metrics were calculated across different frequency bands (theta: 4–7 Hz, alpha: 8–13 Hz, beta: 14–30 Hz, gamma: 31–50 Hz) and conditions (congruent vs. incongruent).

All quantitative analyses were conducted using SPSS version 27.0, employing a significance threshold of *p* < 0.05 as the criterion for statistical significance. Post hoc analyses were conducted using the Bonferroni correction. If the assumption of sphericity was not met, the Greenhouse–Geisser correction was used.

## 3. Results

### 3.1. ERP

**Figure 2:**
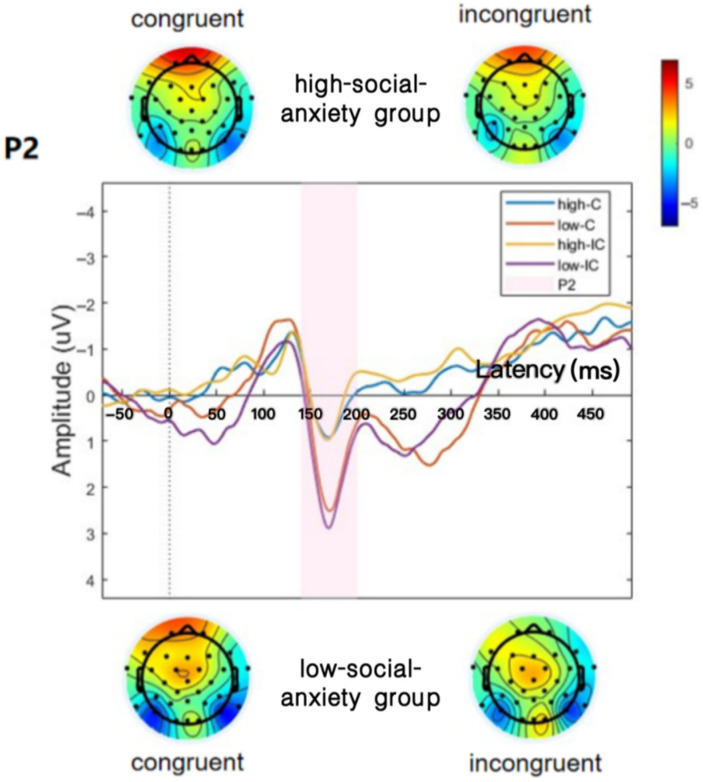
Waveform morphology and scalp topography of P2 components under varying conditions comparing group responses.

The results concerning the P2 component are depicted in Figure 2 and Table 2. The results indicated a significant main effect of group (*F*(1,43) = 4.170, *p* = 0.047, *η_p_*^2^ = 0.088). Participants in the LSA group exhibited significantly greater P2 amplitudes than those in the HSA group (*p* < 0.05). However, no significant main effect of condition was observed (*F*(1,43) = 0.219, *p* = 0.642, *η_p_*^2^ = 0.005). Additionally, the interaction between condition and group was not significant (*F*(1,43) = 1.169, *p* = 0.286, *η_p_*^2^ = 0.026).

A supplementary analysis with age and gender as covariates yielded results consistent with the primary analysis, indicating that neither variable altered the main findings (see Appendix B).

### 3.2. Correlation Analysis

**Figure 3:**
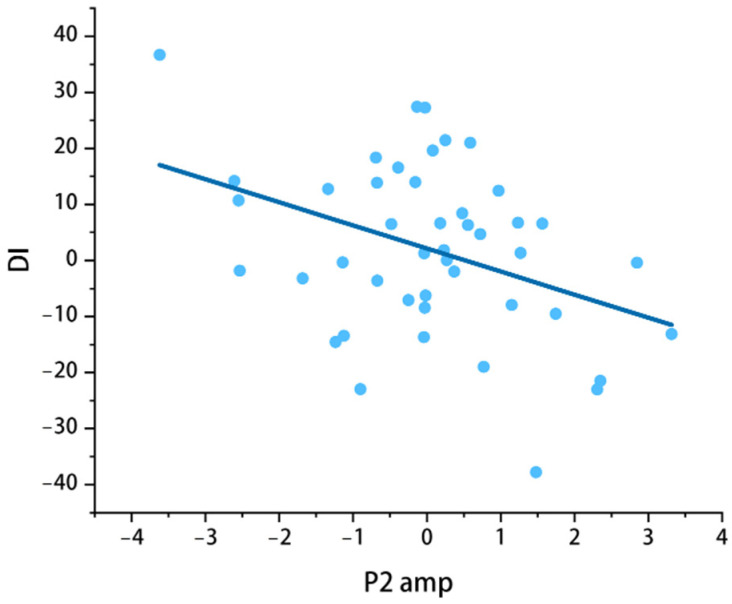
Scatter diagram showing the correlation between P2 amplitude and DI.

Table 3 indicates that there was no significant correlation between social anxiety symptoms and attentional bias (*r_BI_*(43) = 0.220, *r_OI_*(43) = 0.181, *r_DI_*(43) = 0.014, *p* > 0.05). As expected, DI and P2 amp were negatively correlated, *r* = −0.384, *p* < 0.01 (Figure 3).

### 3.3. Functional Connectivity

**Figure 4:**
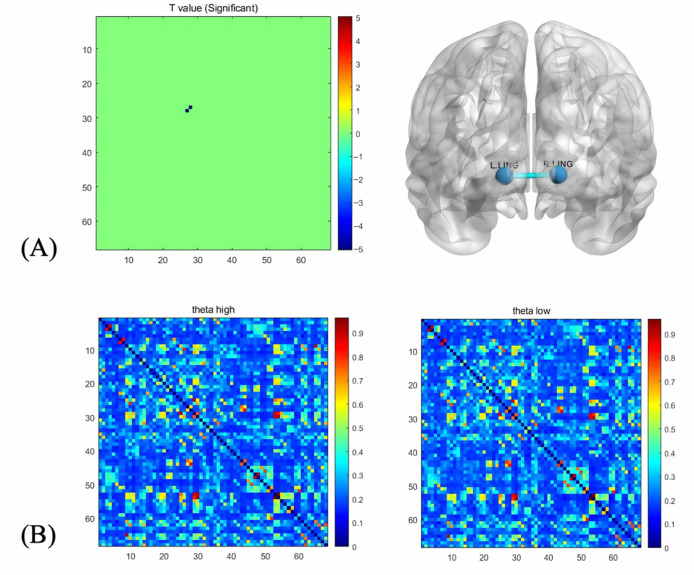
(**A**) Comparison between HSA and LSA under congruent conditions in the theta band. (**B**) Average connectivity for the HSA and the LSA.

Results for the lingual L’–lingual R’ component are depicted in Figure 4. A significant between-group effect was found, *t* = −5.062, *p* < 0.001. In the HSA group, the functional connectivity (*PLV*) was significantly weaker compared to the LSA group (*p* < 0.001). Figure 4 presents the average connectivity for the HSA and LSA.

### 3.4. Graph Theory

(1) Theta

As shown in Table 4, in the congruent condition, results showed that the *Dc* (*t* = 3.721, *p* = 0.039) in the right lateral orbitofrontal in the HSA was significantly higher than in the LSA. However, the *Ne* in the left pericalcarine (*t* = −3.675, *p* = 0.045) in the HSA was significantly lower than in the LSA. In the incongruent condition, the results indicated that the HSA group exhibited significantly higher values than the LSA group in the following measures: *Dc* in the right pars opercularis (*t* = 3.996, *p* = 0.01), *Bc* in the right posterior cingulate (*t* = 4.377, *p* = 0.005), and *Ne* in the right pars opercularis (*t* = 3.857, *p* = 0.026).

(2) Alpha

The results showed that, in comparison to the LSA group, the HSA group had substantially lower *Nlp* in the right lateral orbitofrontal cortex under the congruent condition (*t* = −3.661, *p* = 0.046). In the incongruent condition, the HSA group exhibited significantly higher *Dc* (*t* = 4.005, *p* = 0.016) and *Ne* (*t* = 4.057, *p* = 0.014) in the right lateral orbitofrontal cortex compared to the LSA group. However, the *Nlp* (*t* = −4.074, *p* = 0.013) in the HSA was significantly lower than in the LSA in the same brain region.

(3) Beta

The results showed that the HSA group’s *Bc* in the left banks of the sts was significantly higher than the LSA group’s in the congruent condition (*t* = 4.320, *p* = 0.006). In the incongruent condition, the findings showed that the HSA group exhibited a significantly higher *Dc* in the left parahippocampal region compared to the LSA group (*t* = 3.675, *p* = 0.045).

(4) Gamma

In the incongruent condition, the HSA group exhibited significantly higher *Bc* in the left banks of the superior temporal sulcus (bankssts) compared to the LSA group (*t* = 3.891, *p* = 0.023).

## 4. Discussion

This study employed a combined ERP and functional connectivity approach to examine neural processing of social cues in adolescents with high versus low social anxiety traits. As hypothesized, the high-anxiety group showed a distinct neurophysiological profile characterized by reduced early attentional engagement (P2), attenuated theta-band connectivity in visual-emotional regions, and altered efficiency of brain network nodes. These findings point to differences in attentional control and neural coordination during social information processing in adolescents with high social anxiety traits.

### 4.1. Neural Patterns in Social Information Processing

Adolescence is a critical period of neurological maturation of the brain, involving emotional and social behavior, during which the brain undergoes rapid structural and functional changes, affecting social cognitive abilities (Roberson-Nay & Brown, 2011). This developmental stage constitutes a vulnerable window for social anxiety onset (Edgar et al., 2024). The current study explored social stimulus processing in adolescents with high social anxiety traits, identifying group differences in both ERPs and functional connectivity in cognitive–attentional-related brain regions.

### 4.2. Attentional Processing in Adolescents with High Social Anxiety Traits

The P2 enhancement typically reflects greater sustained, top-down perceptual analysis (Schupp et al., 2003), primarily indexing levels of attentional focus. Greater P2 amplitude may indicate that individuals process stimuli more efficiently and allocate attentional resources more flexibly. In our study, low social anxiety adolescents demonstrated greater P2 amplitude, which may indicate that they are more efficient in cognitive processing when processing social face stimuli. This result is consistent with our hypothesis. In contrast, high-social-anxiety adolescents may show excessive attention to social face stimuli at the early stage of processing, leading to an overconsumption of cognitive resources, which suppresses the P2 amplitude.

### 4.3. Functional Connectivity and Cognitive Efficiency in Adolescents with High Social Anxiety Traits

In terms of functional connectivity, adolescents with high social anxiety traits showed weaker connectivity than those with low social anxiety. These findings did not support our hypothesis. Theta bands (4–7 Hz) are often associated with memory, attention, and emotional processing (Zickerick et al., 2021). The lower *PLV* in the theta band may indicate that adolescents with high social anxiety have difficulties with certain cognitive tasks. The medial occipitotemporal gyrus plays a role in visual information processing and early emotional stimulation (Abdel-Ghaffar et al., 2024). The functional connectivity between the left medial occipitotemporal gyrus and the right medial occipitotemporal cortex in the theta band was lower in adolescents with high social anxiety traits than in those with low social anxiety traits. It can be inferred that high-social-anxiety adolescents are impaired in processing emotionally related visual information. They also require greater mental effort in processing emotional response tasks. Individuals with social anxiety are more likely to have trouble sifting through irrelevant information, according to recent studies (J. Yuan et al., 2021). When socially anxious individuals perceive threatening facial expressions, it becomes more difficult for them to filter out irrelevant information (J. Yuan et al., 2021). This forces them to devote greater cognitive resources to information processing (J. Yuan et al., 2021). This is based on what was found in our study.

### 4.4. Graph Theory Metrics and Brain Network Organization in Adolescents with High Social Anxiety Traits

Graph theory analysis revealed an altered topological organization of brain networks in adolescents with high social anxiety traits. Specifically, the HSA group exhibited increased *Dc* and *Bc* in regions such as the right lateral orbitofrontal cortex and the posterior cingulate. These metrics are associated with a node’s influence and integrative capacity within a network (Rubinov & Sporns, 2010). Elevated values may indicate that these regions become more pivotal hubs for information routing in HSA adolescents, possibly reflecting heightened salience monitoring and compensatory engagement of cognitive control resources when processing social-emotional stimuli (Rubinov & Sporns, 2010). Concurrently, reduced *Nlp* and increased *Ne* in the alpha band were observed. This pattern suggests a shift towards more bilateral and locally integrated processing, which may underlie the difficulty in disengaging from socially salient information, a hallmark of social anxiety (Gilboa-Schechtman & Shachar-Lavie, 2013). The discovery of increased nodal efficiency in the orbitofrontal cortex, a crucial node of the default-mode network (DMN), is noteworthy because it supports new findings that social anxiety is associated with hyper-connectivity in the DMN (Al-Ezzi et al., 2023). Such hyper-connectivity may sustain self-referential processing and threat appraisal, thereby perpetuating anxious states during social perception.

### 4.5. Attentional Bias and Early Emotional Processing in Adolescents with High Social Anxiety Traits

Findings of our study showed that the amplitudes of DI and P2 were negatively correlated. This may imply the possible association between attentional bias and early emotional processing. Large DI means that adolescents are more likely to allocate attention to faces. Low P2 amplitude could mean that this bias makes it harder for them to process facial emotions quickly.

Trujillo et al. (2021) found that when individuals are more focused on emotional cues, they are less efficient at processing emotions early on. This finding is in line with what we found in our study: when adolescents pay more attention to social faces, they are less able to understand facial emotions swiftly. This could therefore cause their later mechanisms for recognizing and responding to emotions to be late or wrong. Studies have found that adolescents with high levels of social anxiety experience difficulties in the early stages of emotional processing (Wauthia et al., 2022a). Under conditions of reduced processing efficiency, they may exhibit more avoidant behaviors or inappropriate reactions in social interactions.

### 4.6. Strengths and Practical Implications

This study has key methodological strengths, including its multi-method neurophysiological approach and its focus on adolescents using an ecologically relevant social cue task. These features enhance the validity of our finding of a neural signature comprising early attentional reduction, compensatory control, and altered network efficiency. Practically, these neural patterns could contribute to the development of biomarkers for risk detection and underscore the need for interventions that target attentional control and cognitive flexibility in adolescents with high social anxiety traits.

### 4.7. Limitation and Future Direction

Our study’s limitations should also be mentioned. First, we cannot prove causation because of our study’s cross-sectional nature. While distinct patterns of ERP components and functional connectivity emerged between HSA and LSA, whether these neural differences precede or result from anxiety symptoms requires further investigation. Second, it is important to emphasize that our participant groups were defined based on self-reported levels of social anxiety traits, rather than by formal clinical diagnosis. Consequently, our findings characterize the neural correlates of high social anxiety traits in adolescents and should be interpreted within this dimensional framework, with caution in generalizing to clinically diagnosed populations. Third, future studies might consider expanding the sample size and utilizing a longitudinal research design to further investigate the social information processing characteristics in adolescents with high social anxiety traits. Moreover, incorporating different types of measures (e.g., eye-tracking) could help to assess the attentional bias and explore its relationship with social anxiety. Fourth, we did not formally assess general cognitive ability (e.g., via IQ tests) or collect detailed developmental histories. Future studies incorporating such measures could help clarify the specificity of the observed neural correlates to social anxiety, independent of broader cognitive factors. Fifth, while we collected information on family structure (e.g., only-child status), this variable was not included in the formal analysis. Future research could explore how such familial factors interact with the neurocognitive mechanisms of social anxiety, providing a more comprehensive developmental perspective. Finally, despite the gender imbalance in our sample, supplementary analysis confirmed it did not affect the main findings (see Appendix B). Future studies with balanced samples are needed.

## 5. Conclusions

Our findings demonstrate that neurophysiological responses during social information processing differ significantly among adolescents with high social anxiety traits. These patterns are characterized by reduced P2 amplitudes and changes in functional connectivity and graph theory metrics during the processing and regulation of facial information. In particular, these adolescents exhibit improved connections across several brain regions as well as modified measures, including node degree and betweenness centrality alterations, as well as increased global efficiency and decreased local efficiency. These findings suggest that adolescents with high social anxiety traits may face challenges in efficiently processing and regulating their attention to social stimuli, which could contribute to the maintenance of social anxiety symptoms. These findings not only advance our neurobiological understanding but also carry practical implications. 

## Figures and Tables

**Table 1:** Demographic characteristics of the HSA and LSA groups.

		HSA (*n* = 27)	LSA (*n* = 18)
Gender	Female	16	4
	Male	11	14
Age (*M* ± *SD*)		15.89 ± 1.41	14.61 ± 3.54
Parental Education Level	Middle School	3	8
	High School	24	10
Only-Child Status		7	4

**Table 2:** Mean amplitudes of components across conditions.

		HSA		LSA						
		*M*	*SD*	*M*	*SD*	*t*	*p*	*d*	95% CI	
P2	Congruent	0.330	1.841	1.625	2.218	−1.578	0.122	−0.480	−2.186	0.267
	Incongruent	0.197	1.860	1.625	2.416	−2.237	0.031 *	−0.681	−2.715	−0.141

Note. * *p* < 0.05.

**Table 3:** Bivariate relationships among attentional bias indices, social anxiety scores, and ERP amplitudes.

		1	2	3	4	5
Behavioral Measures	1 BI	1				
	2 OI	0.614 **	1			
	3 DI	0.316 *	−0.555 **	1		
	4 social anxiety	0.220	0.181	0.014	1	
ERPs	5 P2amp	−0.218	0.127	−0.384 **	−0.143	1

Note. BI = bias index, OI = orienting index, DI = disengagement index; P2amp = P2 amplitude; * *p* < 0.05, ** *p* < 0.01.

**Table 4:** Nodal metrics data across frequency bands.

		Theta		Alpha		Beta		Gamma
		Congruent	Incongruent	Congruent	Incongruent	Congruent	Incongruent	Incongruent
Nodal Metrics	*Dc*	3.721 *	3.996 *	-	4.005 *	-	3.675 *	-
	*Bc*	-	4.377 **	-	-	4.320 **	-	3.891 *
	*Nlp*	-	-	−3.661 *	−4.074 *	-	-	-
	*Ne*	−3.675 *	3.857 *	-	4.057 *	-	-	-

Note. *Dc* = degree centrality, *Bc* = betweenness centrality, *Nlp* = node load proportion, *Ne* = network efficiency; * *p* < 0.05, ** *p* < 0.01.

## Data Availability

The data presented in this study are openly available in Attentional Impairments and Neural Compensation in Socially Anxious Adolescents: A Combined ERP and Functional Connectivity Study at https://pan.baidu.com/s/1Skm0pxnxcuIFsFdLxI5uAA?pwd=bebt, accessed on 19 March 2026 (extraction code: bebt).

## Abbreviations

The following abbreviations are used in this manuscript:

HSA	High Social Anxiety
LSA	Low Social Anxiety
BI	Bias Index
OI	Orienting Index
DI	Disengagement Index
*Dc*	Degree Centrality
*Bc*	Betweenness Centrality
*Nlp*	Node Load Proportion
*Ne*	Network Efficiency

## Appendix A. Behavioral Results

Task performance was calculated by attentional bias (AB) with the reaction time (RT). Attention bias (AB) is an automatic tendency to move towards or away from emotional stimuli (Kruijt et al., 2019). RT was assessed from when the stimulus began until the subject’s response. All tests with RT that differed more than ±2.5 standard deviations from the mean were excluded from analysis to enhance the reliability of the data. To determine the attentional bias (AB) in all the subjects for each group, the disconnection index (DI), orientation index (OI) and bias index (BI) were calculated (Grafton & MacLeod, 2014, 2016; Hornung et al., 2019). Previous studies have validated the construct validity and reliability of these indices, which researchers typically use in the measurement of attention differences, notably in social anxiety and the attention fixation paradigm (Grafton & MacLeod, 2014, 2016; Hornung et al., 2019). These indices provided valid quantitative measures for determining differences in the task performance of different groups in the present study.

AB scores are often obtained by computing differences between reaction time (RT) under neutral and emotional conditions. A positive score indicates a higher approach tendency towards emotional stimuli, while a negative score indicates withdrawal from emotional stimuli (Chen et al., 2012; Thoern et al., 2016).

Subtracting the reaction time (RT) for the congruent and neutral conditions yields the so-called IO. The result reflects initial attention to emotion stimuli (Thoern et al., 2016). A positive IO value indicates that emotional stimuli capture and sustain more attention. The occurrence of a negative IO value indicates that attention is sustained at the same level as that to non-emotion stimuli, as indicated in Y. Wang et al.’s (2024) study.

Disengagement index (DI) is the ability to disengage attention from affective stimuli, as described in the research by Pintzinger et al. (2016). The index is measured based on the difference in variability of the reaction time to irrelevant and neutral stimuli. Y. Wang et al. (2024) note that people with positive DI values fail to disengage attention from emotional material, while people with negative DI values succeed.

RTs and attention bias (disconnection index (DI), orientation index (OI) and bias index (BI)) were analyzed with separate repeated-measures ANOVAs in a 2 (group: HSA vs. LSA) × 2 (condition: congruent vs. incongruent) design.

Results of the RTs showed that there was no significant main effect of condition, *F*(1,43) = 0.584, *p* = 0.449, *η_p_*^2^ = 0.013. There was also no significant main effect of group, *F*(1,43) = 1.031, *p* = 0.316, *η_p_*^2^ = 0.023. The condition × group interaction was nonsignificant, producing *F*(1,43) < 0.00, *p* = 0.993, *η_p_*^2^ < 0.001.

Results of the attention bias indicated that there was no significant main effect of group. *t*_BI_(*43*) = 0.814, *p*_BI_ = 0.420, *d*_BI_ = 0.248; *t*_OI_(*43*) = 0.016, *p*_OI_ = 0.987, *d*_OI_ = 0.005; *t*_DI_(*43*) = 0.841, *p*_DI_ = 0.405, *d*_DI_ = 0.256.

**Table A1:** Average RTs and attention bias between different groups.

	HSA		LSA					
	*M*	*SD*	*M*	*SD*	*t*	*P*	95% CI	*t*
RTs in the congruent conditions	365.876	76.613	368.147	17.186	−1.042	0.303	−78.185	−1.042
RTs in the incongruent conditions	368.147	79.273	394.737	102.758	−0.979	0.333	−81.389	−0.979
BI	2.272	11.946	−1.7341	21.074	0.814	0.420	−5.925	0.814
OI	−1.224	16.814	−1.316	21.056	0.016	0.987	−11.326	0.016
DI	3.494	14.380	−0.434	16.740	0.841	0.405	−5.496	0.841

Note. BI = bias index, OI = orienting index, DI = disengagement index.

## Appendix B. Results of ERP Analysis with Gender and Age as Covariates

To control for the potential influence of gender and age on the research outcomes, both variables were included as covariates in the repeated-measures analysis of variance. The results indicated no significant effect (*ps* > 0.05), suggesting that neither gender nor age exerted a significant influence on the ERP in the study.

**Table A2:** Results of repeated-measures ANOVA with covariates.

		*F*	*p*	*η_p_* ^2^
P2	gender	1.314	0.258	0.030
	age	0.259	0.614	0.006
